# Skin Lightening Practice and Its Cutaneous Effects Among Women in Kinondoni Municipality, Dar es Salaam, Tanzania

**DOI:** 10.1111/jocd.70453

**Published:** 2025-09-17

**Authors:** Msangi Yesaya Francis, Eliaichi Minja, Magdalena Dennis, Grace Shayo

**Affiliations:** ^1^ Muhimbili University of Health Sciences Dar es Salaam Tanzania; ^2^ Muhimbili National Hospital Dar es salaam Tanzania

## Abstract

**Background:**

Individuals who practice skin lightening (SL) may develop various skin conditions, including permanent hyperpigmentation. Despite these adverse effects, people often continue using SL products in an attempt to treat the resulting hyperpigmentation, thereby creating a vicious cycle. The burden of SL among women is unknown in Tanzania.

**Study Aim:**

This study aimed to describe the practice of SL and its cutaneous effects among adult women in Kinondoni Municipality, Dar es Salaam.

**Methodology:**

A cross‐sectional study was conducted from September 2023 to January 2024, using multistage sampling to select households and participants. Consenting members from 611 households were interviewed and examined. SL was identified through self‐reported use of dermo‐cosmetic products, verification of ingredients, and evidence of skin changes. Chi square test and Poisson regression were used to assess factors associated with SL practice. Statistical significance was set at *p* ≤ 0.05.

**Results:**

A total of 1192 women with a median (IQR) age of 32 (26–42) were recruited. About half (52.3%) were married, and 46% had attained primary‐level education. The overall prevalence of SL was 54.5%, with significantly higher prevalence among front desk workers (19%, *p* < 0.048) and petty traders (11%, *p* < 0.03). The most frequently used lightening agents were kojic acid (50%) and hydroquinone (34%). The majority (82%) of women practiced SL for beautification purposes, while only 1.3% used it with a medical prescription. Recommendations from spouses, friends, relatives, and cosmetic vendors, as well as product branding, affordability, and advertising significantly increased the likelihood of practicing SL (all *p* < 0.05).

**Conclusion:**

One in every two women in Kinondoni District was using SL products. The practice was common among front desk workers and petty traders, often following recommendations from friends, relatives, or cosmetic vendors. Health education on the dangers of hazardous cosmetics, along with stricter regulatory control of these products, may help reduce their usage.

## 1 Background

Although an obsession with fair skin is more prevalent among dark‐skinned populations and cultures, the use of skin lightening (SL) products is a global phenomenon. The World Health Organization (WHO) estimates that regular use of skin whitening/lightening cosmetics among women is approximately 40% in China, 61% in India, and 77% in Nigeria [1]. Among African populations, the prevalence of skin lightening ranges from 25% to 67% [2].

SL refers to the application of chemical substances to the skin with the intent to whiten it by reducing melanin concentration. Common agents include kojic acid, ascorbic acid, and retinoic acid [3]. In contrast, skin bleaching involves the use of much harsher and potentially more dangerous chemical agents to whiten the skin beyond its natural color [4]. Skin bleaching is considered an aggressive practice due to the intensity of the techniques and substances used [5]. Chemicals such as mercury iodide, washing powder, sodium hypochlorite (commonly known as bleach), and glutathione injections or pills are used in skin bleaching and can corrode the skin and cause systemic side effects [6]. Due to the serious health risks associated with these products, several countries have banned their use [7]. Skin lightening practices vary widely between countries, with some exhibiting alarmingly high prevalence rates. Studies consistently show that women have higher rates of SL than men, and in some cases, women apply these products to their children as well [8]. In Tanzania, the prevalence and cutaneous effects of SL among the general female population are not well documented. One study conducted in 2011 in Zanzibar, a Tanzanian archipelago in the Indian Ocean, reported a SL prevalence of 18% among commercial sex workers [9].

SL may be initiated either on medical grounds, under the guidance of a physician or dermatologist, or for non‐medical reasons. A study in the United States found that 46% of participants used SL creams as prescribed treatments for hyperpigmentation disorders, with 61% of those cases attributed to melasma [8]. Exploring motivations behind the use of SL products, a study in Malaysia reported that over 53% of participants believed that lighter skin improved self‐confidence and self‐esteem, while 52% perceived it as more attractive, appealing, and healthy [10].

The range of SL products used is extensive and, in some cases, includes steroids. A study in India found that 46% of women practiced SL, and among them, 74% used topical steroids to achieve a fairer complexion. Alarmingly, only 4% had received a prescription for these steroids, and many continued to use these products even after the initial skin condition had resolved [11].

Prolonged use of harmful cosmetic substances can lead to severe complications and, in some cases, fatal outcomes. Common adverse effects include hypopigmentation, hyperpigmentation (especially exogenous ochronosis), erythema, skin thinning, hirsutism, striae, acne, paresthesia, delayed wound healing, and increased susceptibility to infections [12].

## 2 Methods

### 2.1 Study Design, Setting and Population

A cross‐sectional, community‐based study was conducted in 10 of the 20 wards in Kinondoni Municipal District, Dar es Salaam, Tanzania. Kinondoni District was purposefully selected from the five municipal districts of Dar es Salaam, which include Temeke, Kigamboni, Ubungo, Ilala, and Kinondoni itself. Kinondoni municipality is largely urban and peri‐urban (531 km^2^) has the highest population density and the largest number of residents of all the five districts of Dar es Salaam city. Approximately 270 km^2^ out of 531 km^2^ total area is densely built up or urban portion. According to the 2022 National Census, Kinondoni Municipality has a population of 982 328, with a population density of approximately 3645 people per km^2^. Females constitute 51.7% of the residents. Over two‐thirds (69%) of the population are aged between 15 and 64 years. It is estimated that 458 149 residents are employed, with 95% working in the private sector and 5% in the public sector. Additionally, 254 527 individuals are self‐employed while only 3% are involved in subsistence farming in peri‐urban areas [13].

All women meeting the inclusion criteria i.e., women who are residents of (Kinondoni Municipality) aged 18 years or older were recruited.

### 2.2 Participants' Selection

Kinondoni Municipality consists of 20 wards, from which 10 were randomly selected using a pick‐box (lottery) method. Each ward was assigned a number, and the numbers were placed in a box. After thorough mixing, 10 numbers were drawn one by one without replacement. The selected wards included four suburban wards, namely Mbweni, Wazo, Ndugumbi, and Makongo, and six urban wards: Mwananyamala, Kijitonyama, Hananasif, Kinondoni, Kunduchi, and Magomeni. Within each selected ward, all streets were listed, and two streets were randomly selected using the same lottery method. In each selected street, an average of 31 households was systematically selected, starting from the house of the local 50‐cell leader as the reference point. The exact number varied depending on the availability of eligible participants within the households.

A total of 611 households were visited. In each household, all eligible women who met the inclusion criteria were enrolled in the study. We included consenting women aged 18 years or older who were currently residing in Kinondoni Municipality. Participants were recruited through house‐to‐house visits, with an average of approximately 60 participants per street. There were no exclusion criteria.

### 2.3 Interviews and Physical Examination

Participants were interviewed, and their information was documented using a paper‐based questionnaire. They were then asked to present their skin products, and the researchers examined the ingredients listed on the product labels to identify and document any skin lightening or hazardous chemicals present in the formulations.

In addition, while maintaining privacy (with a female research assistant present), the participant was adequately but modestly exposed, and skin examinations were conducted in adequate natural daylight to identify any cutaneous changes associated with the use of skin lightening products. SL was defined by the presence of skin lightening chemicals in the participant's products, along with one or both of the following clinical features: (1) lighter facial skin compared to sun‐protected areas, and/or (2) diffuse lightening of the hands with islands of normal pigmentation over the joints.

Digital photographs of skin lesions were taken from all consenting participants. These images were subsequently reviewed by MYF and two dermatologists (MD and EM), who independently assessed and documented the diagnoses. An agreed diagnosis was defined as one that matched across all three evaluators. In cases of diagnostic disagreement, the three clinicians discussed the findings and reached a consensus diagnosis.

### 2.4 Statistical Analysis

Data analysis was performed using SPSS version 23.0. Categorical variables were summarized as percentages and compared using the Chi‐square test. Modified Poisson regression was employed to determine factors associated with skin lightening practices. Variables with a *p*‐value ≤ 0.2 in the univariate analysis were included in the multivariate model. Statistical significance was set at a *p*‐value ≤ 0.05.

## 3 Results

A total of 611 households were selected, from which 1200 women met the inclusion criteria. Eight women did not consent to participate. As a result, 1192 women were recruited and included in the study.

In this study, over half of the participants (58.9%) were aged between 18 and 35 years, with a median (IQR) age of 32 (26–42) years. More than half (52.3%) were married. Participants with primary and secondary levels of education accounted for 46.3% and 42.4%, respectively. The majority of participants (46%) were engaged in petty trading, while about one‐third were housewives. Each of the 10 wards in Kinondoni Municipality contributed approximately 10% of the participants (Table 1).

**TABLE 1:** Socio‐demographic characteristics of the study participants, *N* = 1192.

Variable	Frequency (*n*)	Percentage (%)
Age group (years)		
18–35	701	58.9
36–45	296	24.7
> 45	195	16.4
Marital status		
Single	447	37.5
Married	624	52.3
Divorced	47	3.9
Separated	24	2.0
Widowed	50	4.2
Level of education		
No formal education	23	1.9
Primary education	552	46.3
Secondary education	506	42.4
College/University	111	9.3
Occupation		
House wives	397	33.3
Front desks^a^	19	1.6
Hospitality personnel^b^	54	4.5
Sales personnel	19	1.6
Entrepreneur/Petty traders	548	46.0
Corporate officials	58	4.9
Student	64	5.4
Others	33	2.8
Residence (Wards)		
Makongo	119	10.0
Mwananyamala	120	10.1
Wazo	118	9.9
Ndugumbi	120	10.1
Magomen	119	10.0
Mbweni	111	9.3
Hananasifu	119	10.0
Kinondoni	125	10.5
Kijitonyama	120	10.1
Kunduchi	121	10.2

^a^ Front desk workers included: secretaries, receptionists, air hostesses, and bank tellers.

^b^ Hospitality personnel included: barmaids and hoteliers.

**FIGURE 1:**
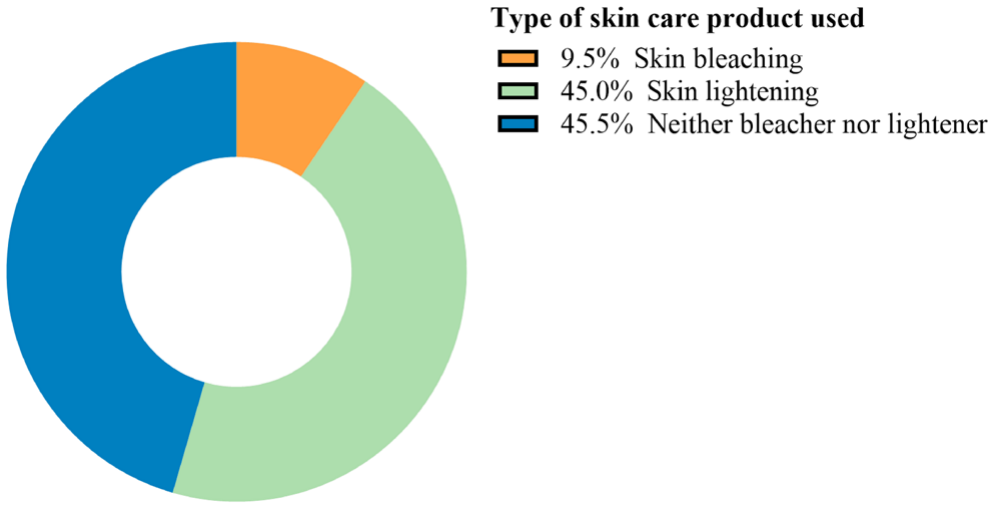
Magnitude of skin lightening practice and type of SL products among participants.

Over half of the participants (632/1192; 54.5%) were practicing SL. Among those who practiced SL (*n* = 632), 9.5% used skin bleachers and 45% used skin lighteners (Figure 1).

A higher proportion of participants (79.9%) who practiced skin lightening used creams, while 30.1% used lotions. The majority (93.0%) reported applying their skin lightening products (SLPs) to both the face and other parts of the body. Most had been using SLPs for over 6 months (69.8%) and applied the products twice daily (53.3%). Over half (53.3%) of the women who practiced skin lightening had type V skin tone (Table 2).

**TABLE 2:** Formulations and trends of application of skin lightening products among the participants *n* = 632.

Variable	Frequency (*n*)	Percent (%)
Type of skin lightening product^a^		
Creams	505	79.9
Lotion	190	30.1
Jellies	29	4.6
Ointments	43	6.8
Pills	3	0.5
Soaps	23	3.6
Others	7	1.1
Area of application		
Face only	44	7.0
Face and body	588	93.0
Duration of use of SLPs		
< 6 months	191	30.2
> 6 months	441	69.8
Current user of SLPs		
Yes	555	87.8
No	77	12.2
Frequency of application of SLPs^b^		
Once a day	218	34.5
Twice a day	337	53.3
Three times a day	63	10.0
Weekly	14	2.2
Natural skin tone of participants		
Type VI	202	31.2
Type V	333	53.3
Type IV	97	15.5

^a^ Percentage exceeds 100 because some participants used more than one formulation of skin lightening products.

^b^ SLPs: Skin lightening products.

**FIGURE 2:**
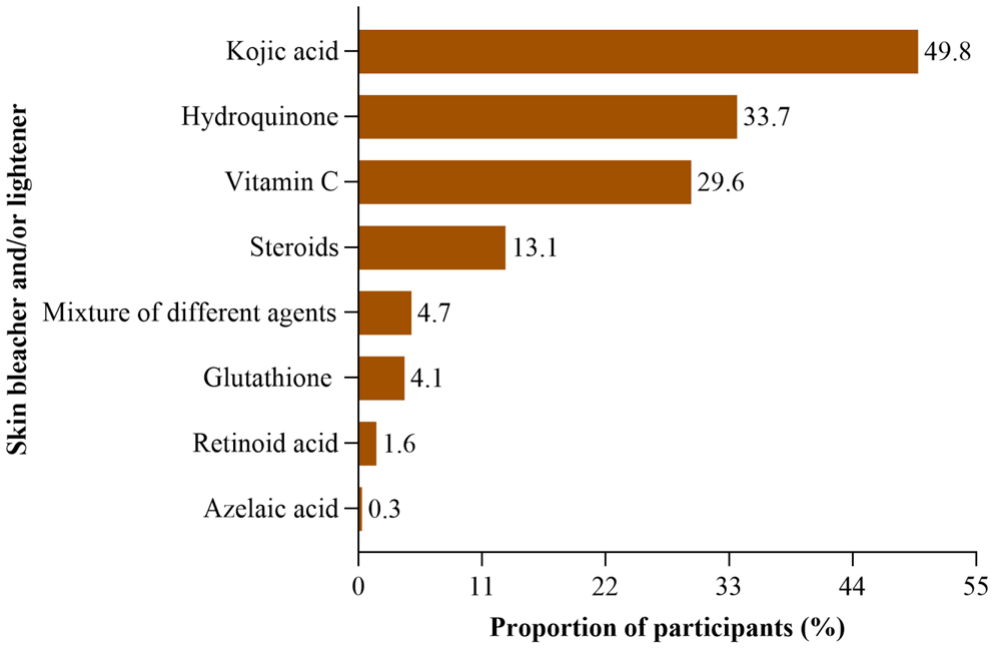
Common skin lightening and bleaching agents listed in packages of skin lightening products used by the women. NB: The percentage sum exceeds 100 because some of the SLPs contained more than one lightening agent thus counted more than once.

Half of the inspected skin care products contained Kojic acid as a skin lightening ingredient. Hydroquinone (34%) and Vitamin C (29.6%) were the next most common ingredients. Other chemicals not shown in Figure 2 such as stearic acid, arbutin, whitening oil, carrot oil, silicone oil, and dimethicone were also found in the majority (76.1%) of skin care products used by participants. Some products listed vague ingredients such as “whitening complex,” “lightening oil,” “brighteners,” or “fruit oil,” without specifying the exact chemical components (Figure 2).

Six of the products had no listed ingredients. Homemade products were reported to consist mainly of mixtures of various agents, including washing powder, sodium hypochlorite, mercury‐containing soaps, local herbs, alkaline soaps, vinegar, unidentified creams, and pills. These were combined and stirred to form a soft mixture of skin bleaching product with unknown concentrations of each ingredient.

Of the 632 respondents who practiced SL, 525 participants (83.9%) reported that they did not read the ingredients list on skin lightening products before purchasing them, primarily due to a lack of knowledge about chemical components. The majority of participants (87.5%) used skin lightening products for beauty reasons, while 19.8% used them in an attempt to treat rashes without a doctor's prescription. A small proportion used SL products to improve social status (8.2%), satisfy their spouse (6.0%), or based on medical prescriptions (1.7%). In exploring the reasons for initiating SL practice, nearly 60% (59.3%) of those who practiced SL reported being influenced by friends or relatives. Other reasons included the affordability of the products (34.2%), perceived effectiveness over a short period (22.9%), brand influence (20.1%), widespread advertising, particularly on social media (8.1%), spouse encouragement (4.7%), perceived safety (5.9%), and approval by regulatory authorities (0.3%) (Table 3).

**TABLE 3:** Modified Poisson regression of factors associated with skin lightening practice among women at Kinondoni Municipality, *N* = 1192.

Variable	Category	Univariate analysis OR (95% CI)	*p* value	Multivariate analysis OR (95% CI)	*p* value
Age (years)	18–35	1.25 (1.05–1.48)	0.013	1.08 (0.94–1.23)	0.276
	36–45	1.37 (1.14–1.65)	0.001	1.02 (0.88–1.18)	0.795
	> 45	Ref			
Education	No formal	0.59 (0.32–1.11)	0.104		
	Primary	0.95 (0.79–1.15)	0.615		
	Secondary	0.97 (0.99–1.20)	0.972		
	College/University	Ref			
Occupation	Student	0.97 (0.73–1.30)	0.844	1.01 (0.80–1.28)	0.905
	Corporate officials	1.06 (0.80–1.39)	0.705	1.02 (0.82–1.29)	0.835
	Entrepreneur	1.25 (1.10–1.42)	0.001	1.11 (1.01–1.23)	0.030
	Sales personnel	1.64 (1.26–2.15)	< 0.001	1.21 (0.94–1.56)	0.131
	Hospitality	1.31 (1.04–1.66)	0.023	1.06 (0.89–1.26)	0.548
	Front desks	1.49 (1.08–2.06)	0.016	1.34 (1.00–1.79)	0.048
	Other	1.02 (0.71–1.45)	0.902	0.98 (0.76–1.27)	0.876
	House wives	Ref			
Skin tone	Type IV	1.00 (0.85–1.19)	0.972		
	Type V	1.07 (0.95–1.21)	0.256		
	Type VI	Ref			
Product advertisement	Yes	1.51 (1.32–1.73)	< 0.001	1.28 (1.10–1.48)	0.001
	No	Ref			
Friend's recommendation	Yes	2.83 (2.56–3.14)	< 0.001	1.53 (1.37–1.71)	< 0.001
	No	Ref			
Concerned about ingredients in the products	Yes	1.39 (1.17–1.66)	< 0.001	1.26 (1.03–1.54)	0.022
	No	Ref			
Affordability of the product	Yes	1.55 (1.40–1.71)	< 0.001	1.21 (1.11–1.31)	< 0.001
	No	Ref			
Brand influence	Yes	1.91 (1.78–2.06)	< 0.001	1.19 (1.08–1.30)	0.001
	No	Ref			
Spouse's influence	Yes	1.88 (1.78–1.99)	< 0.001	1.33 (1.11–1.60)	0.002
	No	Ref			
Effectiveness of the product	Yes	2.02 (1.89–2.17)	< 0.001	1.39 (1.26–1.52)	< 0.001
	No	Ref			
Long‐term safety of the product	Yes	0.86 (0.68–1.10)	0.233		
	No	Ref			
Approval by regulatory authorities	Yes	0.52 (0.16–1.69)	0.280		
	No	Ref			
Own choice	Yes	0.91 (0.82–1.01)	0.065	0.98 (0.90–1.06)	0.553
	No	Ref			
Pharmacy dispensers' recommendation	Yes	1.29 (0.97–1.73)	0.084	1.43 (1.06–1.94)	0.020
	No	Ref			
Medical prescription	Yes	1.34 (0.93–1.93)	0.115	1.98 (1.29–3.03)	0.002
	No	Ref			
Relative and friend's recommendation	Yes	2.93 (2.62–3.27)	< 0.001	1.80 (1.59–2.04)	< 0.001
	No	Ref			
Beauticians/Cosmetics seller's recommendation	Yes	2.43 (2.22–2.66)	< 0.001	1.54 (1.41–1.67)	< 0.001
	No	Ref			

Regarding recommendations on the type of SL products used, about two‐thirds (64.2%) of participants stated that their products were recommended by friends or relatives.

Entrepreneurs and petty traders had an 11% increased likelihood of practicing SL compared to housewives (aPR = 1.11, 95% CI: 1.01–1.23, *p* = 0.03). Similarly, front desk workers had a 19% higher likelihood of practicing SL than housewives (aPR = 1.19, 95% CI: 1.00–1.79, *p* = 0.048). Age, marital status, and education levels were not found to be significant predictors of SL product use. Participants who had been exposed to widespread advertisements of SL products via social media platforms, television, magazines, or posters had a 28% increased likelihood of practicing SL compared to those who had not (aPR = 1.28, 95% CI: 1.10–1.48, *p* = 0.001). Those who considered affordability a key factor had a 55% higher likelihood of practicing SL than those unconcerned about price (aPR = 1.55, 95% CI: 1.11–1.31, *p* = 0.001). Participants influenced by specific brands were 19% more likely to engage in SL practices than those indifferent to branding (aPR = 1.19, 95% CI: 1.08–1.30, *p* = 0.001). Additionally, those persuaded by relatives or friends had an 80% increased likelihood of practicing SL compared to those who were not (aPR = 1.80, 95% CI: 1.59–2.04, *p* = 0.001). Furthermore, participants who received SL product recommendations from cosmetic vendors or beauticians were 54% more likely to practice SL than those who did not (aPR = 1.54, 95% CI: 1.41–1.67, *p* < 0.001) (Table 3).

**FIGURE 3:**
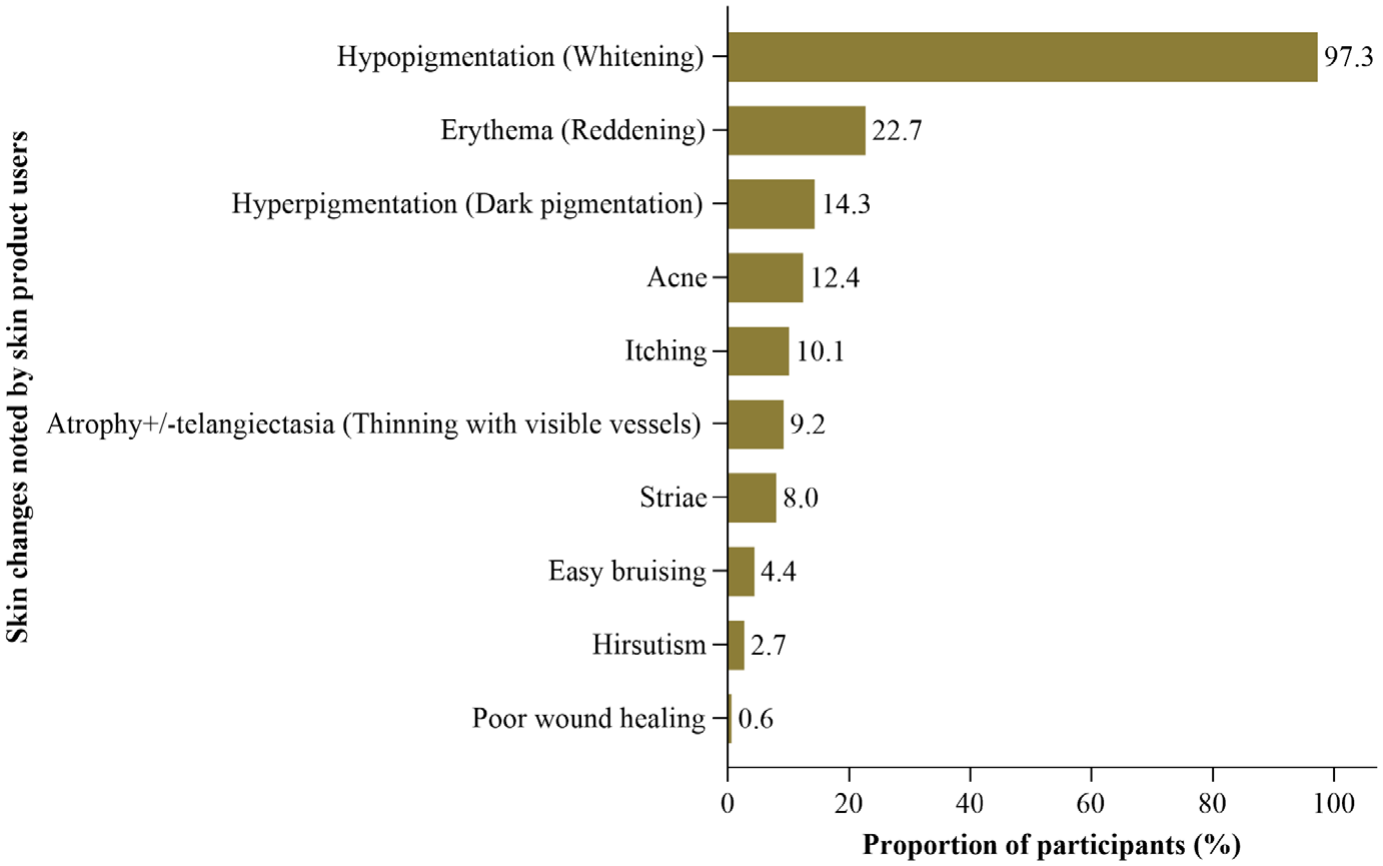
Cutaneous conditions found in women who practiced skin lightening.

Of the 632 participants who practiced skin lightening, 524 were diagnosed with various skin conditions related to the use of skin lightening products. In order of prevalence, these conditions included hypopigmentation (97.3%), erythema (22.7%), hyperpigmentation (14%), acne (12.4%), pruritus (10.1%), telangiectasia or skin atrophy (9.2%), striae (8.0%), easy bruising (4.4%), hirsutism (2.7%), and poor wound healing (0.6%) (Figure 3).

## 4 Discussion

This study revealed three significant findings worth noting. Firstly, the prevalence of skin lightening (SL) practices was high among women in Kinondoni Municipality. Secondly, within the study population, occupation, widespread advertising, and recommendations from cosmetic vendors, relatives, spouses, and friends were influential factors associated with SL practices. Additionally, skin product's brand, ingredients, affordability, and its effectiveness were significantly associated with SL practices. Thirdly, the most common adverse skin conditions related to SL were erythema, hyperpigmentation, pruritus, and acne.

The prevalence of SL practice in the present study (54.5%) is comparable to findings in Malaysia (60%) [10], Togo (59%) [2], and Ghana (50%) [14]. However, it was higher than the prevalence reported in a study conducted in Zanzibar, Tanzania (18%) [9], despite the Zanzibar study involving a higher‐risk group: commercial sex workers. This difference may be partly attributed to the time gap between the two studies: the Zanzibar study was conducted in 2011, while the current study took place in 2023. In recent years, there has been an influx of cosmetic products from abroad, as well as increased access to digital platforms such as online shops and social media, making women more vulnerable to SL practices. A study by Razi et al. analyzed 152 fairness cream commercials on YouTube and found that 84.21% targeted female consumers, while only 15.79% targeted male consumers. Over 77% of the commercials featured celebrities, and less than 50% mentioned specific ingredients in their products [15]. This trend shows that the use of SL products is largely influenced by social networks and media rather than autonomous decision‐making. Therefore, describing SL as a matter of personal choice should be approached with caution.

In the present study, nearly 90% of participants reported using SL products for beautification purposes. This aligns with findings from other countries; for instance, a study in Malaysia reported that over half of the respondents believed SL products gave them high self‐esteem, a more appealing appearance, and healthier‐looking skin [10]. In Saudi Arabia, 44.7% of female participants in one study agreed or strongly agreed that a lighter skin tone is more beautiful [16].

Similar to findings from studies conducted in Saudi Arabia [16] and Rwanda, [17] SL practices in the present study were more common among women under 50 years of age, though without statistical significance. Younger participants cited beautification and financial independence as motivations, as they could afford to purchase dermo‐cosmetics. Front desk workers and petty traders were significantly more likely to engage in SL, possibly because these occupations involve frequent public interaction, where appearance may be perceived as important. The lack of significant association between SL use and variables such as education, marital status, age, skin tone, or education level, but external factors like advertising and occupational demands supports the argument that simple public education campaigns are insufficient and that policy‐level interventions such as stricter regulation of product distribution, crackdowns on misleading advertising, and mandatory labeling are urgently needed.

In the present study, SL practices were significantly associated with recommendations from friends, spouses, relatives, beauticians, and pharmacists. More than half of the participants who used SL products reported being influenced by friends, relatives, or cosmetic vendors. This highlights that SL is not merely a cosmetic choice, but is strongly linked to social acceptance and self‐esteem, reflecting broader consumer psychology and sociocultural dynamics. Other motivating factors that significantly influenced SL practices included the affordability of the products and the rapidity with which the products produced visible effects on the skin. Most participants reported purchasing products at prices ranging from 3000 to 10 000 Tanzanian shillings (approximately 1.1 to 3.7 USD at an exchange rate of 1 USD = 2700 Tsh). This differs from findings in Saudi Arabia, where physician prescriptions were the most common influence, reported by 49.3% of respondents [16]. This underscores the importance of community education on the safe use of cosmetics, especially considering that only a small percentage (1.7%) of women in the current study reported receiving advice from doctors or pharmacists.

Among other cutaneous effects such as hypopigmentation and erythema that were reported in this study, hyperpigmentation, including exogenous ochronosis, was also prevalent. This finding is consistent with a study from the United States, which found post‐inflammatory hyperpigmentation to be among common skin conditions among dark‐skinned individuals seeking dermatologic care [18]. The studies from Malaysia and Saudi Arabia have reported side effects such as skin peeling, acne, redness, sun sensitivity, skin cracking, pain, telangiectasia, and hirsutism [10, 16].

The ingredients in SL products vary widely and can have different adverse effects. In this study, most products contained kojic acid, hydroquinone, vitamin C, steroids, and various locally mixed agents commonly referred to as “mkorogo.” These mixtures included creams, mercury‐containing soaps, washing powders, vinegar, bleaching soaps, sulfuric acids, palm oil, local herbs, glutathione, retinoic acid, and azelaic acid. This contrasts with findings from a U.S.‐based study, which reported hydroquinone, corticosteroids, and mercury‐based products as the most commonly used SL ingredients [19].

Although Kojic acid, the most commonly found ingredient in this study, is generally considered safe at concentrations ≤ 2%, the concentration levels were not indicated on most product labels. Several participants experienced adverse effects, suggesting that the products may have contained higher, unsafe concentrations.

Currently in Tanzania, regulation governing the importation and distribution of cosmetics is primarily under the Tanzania Food, Drugs and Cosmetics Act, 2003 (Chapter 219), which grants authority to regulate cosmetics; covering importation, manufacture, distribution, labeling, sale, and premises registration. Regulations under this Act include the Tanzania Food, Drugs and Cosmetics (Control of Cosmetics) Regulations, 2010, and other relevant subsidiary legislation [20]. Since 1 July 2019, the responsibilities for regulating cosmetics (and food) were moved from TFDA (now TMDA) to the Tanzania Bureau of Standards (TBS). TBS now handles product registration (local & imported), premises inspection and licensing, import/export permit issuance, postmarket surveillance, testing, analysis, compliance monitoring, and public education [21]. Cosmetics must not contain prohibited or harmful ingredients as listed under the Act; only approved colors and substances may be used. The Minister may issue prohibitions via official Gazette notices based on advice from the Director‐General. Offenders face fines ranging from 5 million to 50 million TZS for first‐time violations and up to 100 million TZS and possible criminal prosecution for repeat offenses or serious infractions like selling mercury‐ or hydroquinone‐laden products [22]. Despite strong legal frameworks, Tanzania faces issues with enforcement fragmentation, limited technical capacity, and market non‐compliance especially from unregistered sellers and e‐commerce platforms. This highlights the need for stricter regulations to prevent unlabeled products from entering the country.

## 5 Study Limitations

Seventeen participants were unable to present the skin care products they were using. Instead, they recalled the products they were currently using. This might have introduced a recall bias in the study.

The skin changes observed in the women might have been the result of different products used in the past, and not necessarily those currently in use.

## 6 Conclusion

Skin lightening practice is very common among women in Kinondoni Municipality. Most women were persuaded to use skin lightening products by relatives, friends, their occupation, or cosmetic vendors and were unaware of the presence of hazardous chemicals in these products. Many developed unwanted skin conditions, including permanent hyperpigmentation. Mass health education about the dangers of hazardous cosmetics may help reduce the prevalence of skin lightening practices.

## Conflicts of Interest

The authors declare no conflicts of interest.

## Data Availability

The data that support the findings of this study are available on request from the corresponding author. The data are not publicly available due to privacy or ethical restrictions.
